# Combined Vitamin E and selenium supplementation enhances antioxidant status, reduces disease incidence, and improves economic returns in transition dairy cows

**DOI:** 10.14202/vetworld.2025.2439-2449

**Published:** 2025-08-26

**Authors:** Yixuan Ding, Rui Sun, Xuejie Jiang, Yu Hao, Yuxi Song, Xiaochen Jia, Yunlong Bai, Cheng Xia

**Affiliations:** 1 Department of Clinical Veterinary Medicine, College of Animal Science and Veterinary Medicine, Heilongjiang Bayi Agricultural University, Daqing 163319, China; 2 General Dairy Farming Professional Cooperative of Mudanjiang Agricultural Reclamation Area, Heilongjiang Province, China

**Keywords:** dairy cows, disease prediction, economic benefit, milk yield, oxidative stress, selenium, transition period, Vitamin E

## Abstract

**Background and Aim:**

Dairy cows in the transition period are highly vulnerable to oxidative stress and immune suppression, which increases the risk of metabolic and infectious diseases. Vitamin E (VE) and selenium (Se) are essential antioxidants known to mitigate these challenges, but their combined effects remain underexplored in transition cows. This study aimed to evaluate the effects of VE and Se supplementation–individually and in combination–on oxidative stress biomarkers, immune function, disease incidence, reproductive performance, and economic outcomes in transition dairy cows.

**Materials and Methods:**

Forty Holstein cows with similar baseline characteristics were randomly assigned to four groups (n = 10 each): Control (basal diet), VE (3,000 IU/head injected on days 7 and 14 postpartum), Se (1.5 mg/kg body weight orally from calving), and VE + Se (both interventions). Blood samples were collected on calving day and at 7, 14, and 21 days postpartum. Parameters assessed included non-esterified fatty acid (NEFA), β-hydroxybutyrate (BHB), aspartate aminotransferase (AST), blood urea nitrogen, total antioxidant capacity (T-AOC), superoxide dismutase (SOD), glutathione peroxidase (GSH-Px), malondialdehyde (MDA), interleukin (IL)-1β, IL-6, haptoglobin (HP), milk yield, disease incidence, and economic performance. Receiver operating characteristic (ROC) curves assessed VE and Se’s predictive value for disease.

**Results:**

Combined VE + Se supplementation significantly increased plasma VE and Se levels and improved antioxidant capacity (↑T-AOC, SOD, GSH-Px; ↓MDA) and immune markers (↓IL-1β, IL-6, HP). NEFA and BHB were reduced without affecting AST. The VE + Se group showed significantly lower incidences of mastitis, metritis, and ketosis (p < 0.05). ROC analysis demonstrated high predictive value of plasma VE and Se for disease risk (area under the curve up to 0.973). Economic analysis showed the highest net profit (¥111.91/day) in the VE + Se group.

**Conclusion:**

Combined VE and Se supplementation during the transition period enhances antioxidant and immune function, reduces metabolic disease incidence, and improves productivity and profitability in dairy cows. These findings support integrated micronutrient strategies for periparturient health management. Larger-scale and long-term studies are recommended to confirm these outcomes and explore underlying mechanisms.

## INTRODUCTION

The transition period is typically defined as the interval from 3 weeks pre-partum to 3 weeks post-partum [1]. During this period, dairy cows experience immunosuppression and are prone to oxidative stress, both of which contribute to an unfavorable energy balance [2]. Oxidative stress results in the overproduction of reactive oxygen species, which can exceed the capacity of endogenous antioxidant defense mechanisms, leading to their accumulation in peripheral blood and disruption of redox-regulated biological processes. Concurrently, immunosuppression reduces the number and function of immune cells in cows, thereby increasing the incidence and severity of various diseases [3]. This heightened disease risk during the transition period is associated with reduced milk yield, elevated veterinary costs, and shortened productive lifespan in dairy cows, collectively causing significant economic losses for the dairy industry [4].

Vitamin E (VE) and selenium (Se) are essential micronutrients that function as antioxidants and immune modulators [5, 6]. VE is a fat-soluble vitamin that plays a critical role in dairy cow health [7]. The normal plasma VE concentration in dairy cows ranges from 4.5 mg/L to 6.0 mg/L, with values below 4.0 mg/L indicative of deficiency [8]. Plasma VE levels typically decrease before calving and reach their lowest point after parturition [9]. Several studies have demonstrated that VE supplementation during the transition period significantly reduces markers of oxidative stress such as superoxide dismutase (SOD), malondialdehyde (MDA), and catalase (CAT), while enhancing total antioxidant capacity (T-AOC), granulocyte phagocytic activity, and lymphocyte proliferation activity, thereby alleviating immunosuppression [10, 11]. Administering 1,000–3,000 IU of VE per cow daily during this period has been shown to reduce the risk of intramammary infections and clinical mastitis while improving milk quality [12, 13].

Se is a vital component of glutathione peroxidase (GSH-Px) and plays a pivotal role in maintaining redox balance and animal health [14]. It mitigates oxidative stress by regulating key antioxidant enzymes, including MDA, CAT, SOD, and GSH-Px [15]. Se deficiency impairs neutrophil phagocytic ability in both blood and milk [16]. The standard plasma Se concentration ranges from 51 μg/L to 85 μg/L, and increasing dietary Se intake by 0.5 mg/kg of dry matter (DM) is recommen-ded to meet this standard [17]. Se supplementation has been shown to increase immunoglobulin G levels in serum and colostrum [18], enhance antioxidant capacity [19], improve immune function [20], reduce the incidence of uterine inflammation and ovarian cysts, and lower embryo mortality within the 1^st^ month of pregnancy [16].

Although the individual roles of VE and Se have been extensively studied, recent reviews and meta-analyses have emphasized that their combined and integrative effects on immunity, oxidative stress, and disease resistance during the transition remain insufficiently characterized [21–23].

Despite the well-established roles of VE and Se as individual antioxidants and immune modulators in dairy cows, current literature lacks comprehensive studies that evaluate their combined effects during the transition under practical farming conditions. Most existing studies have focused on either VE or Se alone, overlooking potential synergistic interactions that may provide enhanced protection against oxidative stress and immunosuppression. Moreover, previous trials often varied widely in supplementation protocols, dosages, and assessment parameters, making cross-study comparisons difficult and limiting the development of standardized recommendations. Recent reviews and meta-analyses have acknowledged this limitation, emphasizing the need for integrated research that simultaneously assesses the metabolic, immunological, and productive responses to VE and Se co-supplementation. In addition, while several studies have reported reductions in disease incidence or improvements in milk production following antioxidant supplementation, few have incorporated predictive modeling or economic analyses to evaluate the cost-effectiveness and early diagnostic potential of these micronutrients. The lack of such multidimensional studies creates a significant knowledge gap in the formulation of evidence-based strategies for improving transition cow health and farm profitability.

Therefore, the objective of this study was to systematically investigate the effects of concurrent VE and Se supplementation on oxidative stress biomarkers, immune function indicators, metabolic parameters, production performance, and disease prevalence in dairy cows during the transition period. In addition, the study aimed to evaluate the predictive value of plasma VE and Se concentrations for early identification of common post-partum diseases such as mastitis, metritis, and ketosis using receiver operating characteristic (ROC) curve analysis. Finally, an economic benefit analysis was performed to assess the cost-effectiveness of different supplementation strategies. By addressing both biological efficacy and practical applicability, this study seeks to provide a comprehensive framework for improving periparturient dairy cow management through targeted antioxidant support.

## MATERIALS AND METHODS

### Ethical approval

All experimental procedures involving animals were approved by the Institutional Animal Care and Use Committee of Heilongjiang Bayi Agricultural University (Protocol No. DWKJXY2023057). Cows were housed in an open free-stall barn with unrestricted access to fresh water. Ambient temperatures ranged from −18°C to 15°C; cows were fed a total mixed ration *ad libitum* and were milked 3 times daily.

### Study period and location

This study was conducted from February 2024 to May 2024 at the Experimental Dairy Farm of Heilongjiang Bayi Agricultural University, Daqing, Heilongjiang Province, China (46°35′ N, 125°11′ E; altitude 147 m).

### Animal selection and experimental design

Forty Holstein cows with similar plasma VE and Se levels, parity, body condition scores (BCSs), and average daily milk yields (~30 kg) during the previous lactation were selected. After parturition, cows were randomly assigned to four treatment groups (n = 10 per group):

- Control group: received a basal diet only
- VE group: injected with 3,000 IU VE per head on post-partum days 7 and 14
- Se group: supplemented with 1.50 mg/kg body weight (BW) of Se daily from calving
- VE + Se group: injected with 2010 mg VE (equivalent to 3,000 IU) on days 7 and 14 post-partum and supplemented with 1.50 mg/kg BW of Se from day 0 post-partum.

The VE dosage was selected based on studies demonstrating that high-dose α-tocopherol improves oxidative and immune function in transition cows [21, 22]. The Se dose was based on prior research using organic Se sources shown to be safe and effective [21].

### Housing, feeding, and management

All cows were housed in free-stall barns with *ad libitum* access to water and were milked 3 times daily. Total mixed rations (TMR) formulated for early lactation were provided according to the National Research Council [24] guidelines. The TMR contained:

- 8.5 kg concentrate
- 10 kg silage
- 4.5 kg cottonseed
- 4 kg brewer’s grains
- 3 kg corn flakes
- 5.5 kg soybean meal
- 2 kg molasses
- 7 kg water.

The nutritional composition of the diet was: 59.45% crude fiber, 18.72% crude protein, 2.02% crude fat, 41.08% neutral detergent fiber, and 22.59% acid detergent fiber.

Cows were monitored twice daily for appetite, posture, udder health, and body temperature. Animals diagnosed with mastitis or metritis received immediate treatment according to standard farm veterinary protocols. Cows displaying distress were removed from the trial and treated appropriately.

### Blood sample collection

Blood samples (10 mL) were collected from the tail vein before morning feeding on the day of calving and days 7, 14, and 21 postpartum. Samples were collected into heparinized tubes (Biosharp, Hefei Lanjek Technology Co., Ltd., China), centrifuged at 4,000 × *g* for 5 min, and the supernatant was transferred to 1.5 mL tubes. A second centrifugation at 12,000 × *g* for 5 min was performed under refrigeration. Plasma was stored at −80°C for subsequent analysis. Hemolyzed samples were excluded. All analyses were completed within 30 days, and samples were thawed only once before testing.

### Plasma biochemical and antioxidant analysis

Plasma concentrations of non-esterified fatty acids (NEFA), β-hydroxybutyrate (BHB), blood urea nitrogen (BUN), and aspartate aminotransferase (AST) were determined using a Mindray BS-830S automatic biochemistry analyzer and commercial kits (Mindray Biomedical Electronics Co., Shenzhen, China).

Antioxidant and immune biomarkers were assessed using kits from Nanjing Jiancheng Bioengineering Institute (Nanjing, China):

- Antioxidant markers: T-AOC, SOD, GSH-Px, and MDA
- Immune markers: interleukin-1β (IL-1β), interleukin-6 (IL-6), and haptoglobin (HP).

Plasma VE and Se levels were quantified using commercial kits (Shanghai Uban Biotech Co., Ltd., Shanghai, China).

### Disease definitions

Ketosis was defined as a plasma BHB concentration ≥1.20 mmol/L [25]. Mastitis was diagnosed based on clinical signs, including increased heart rate, anorexia, enlarged udder, abnormal teat structure, and altered milk secretion [26]. Metritis was defined by the presence of a foul-smelling, red–brown vaginal discharge within 21 days postpartum and a rectal temperature >39.5°C. Diagnosis was confirmed through postpartum palpation by trained veterinarians [27].

### Statistical analysis

All statistical analyses were performed using Statistical Package for the Social Sciences 26.0 (IBM, Armonk, NY, USA).

- One-way analysis of variance was used to compare BCS loss among groups
- Repeated measures data (BCS, plasma biomarkers, and milk yield) were analyzed using a mixed-effects model
- 2022;Disease incidence among groups was compared using Chi-square tests with Bonferroni correction for pairwise comparisons
- Spearman’s correlation was used to assess the relationship between VE/Se supplementation (measured as change in plasma levels from day 0 to day 21) and disease outcomes
- Binary logistic regression and ROC curve analysis were employed to evaluate the predictive power of plasma VE and Se levels for disease diagnosis.
- Statistical significance was set at p < 0.05.

## RESULTS

### Baseline characteristics of experimental groups

Table 1 presents the average BCS, milk yield, and parity of the four groups. Each group consisted of 10 cows. No significant differences were observed in parity or BCS among the control, VE, Se, and VE + Se groups. However, milk yield was significantly higher in the VE + Se group compared with the control group (p < 0.05), indicating a potential synergistic effect of combined supplementation on production performance.

**Table 1:** Average body condition scores, milk yields, and parity of the four dairy cow groups.

Item	Groups			

	Control (n = 10)	VE (n = 10)	Se (n = 10)	VE + Se (n = 10)
Parity (times)	2.50 ± 0.53	2.40 ± 0.52	2.50 ± 0.50	2.50 ± 0.51
BCS	3.73 ± 0.30	3.53 ± 0.36	3.55 ± 0.16	3.55±0.20
Milk yield (kg/d)	29.98 ± 8.73^a^	37.83 ± 5.62	36.43 ± 6.17	39.29 ± 6.39^b^

^a and b^indicate significant differences (p < 0.05). VE=Vitamin E, Se=Selenium, BCS=Body condition score

### Plasma VE and Se concentrations

**Figure 1:**
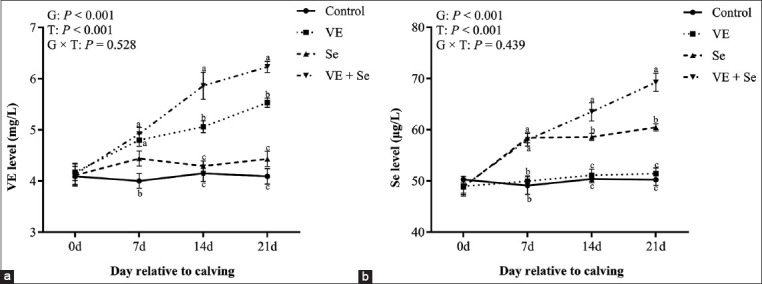
(a) Plasma VE and (b) Se levels in the four experimental groups. Control (n = 10), VE (n = 10), Se (n = 10), and VE + Se (n = 10) groups. Different lowercase letters (a, b, c) indicate statistically significant differences at p < 0.05, T=Time effect, G=Between-group effect, G × T=Inter-group interaction with time, respectively, VE=Vitamin E, Se=Selenium.

Figure 1 displays changes in plasma VE and Se levels over time. Both group and time effects were significant for VE and Se levels (p < 0.001), while the group × time interaction was not significant (p > 0.05). At 7, 14, and 21 days post-partum, plasma VE levels were significantly elevated in the VE and VE + Se groups compared to the control group (p < 0.05), with the highest concentrations in the VE + Se group. Similarly, plasma Se levels were significantly higher in the Se and VE + Se groups (p < 0.05), again with the VE + Se group showing the highest values.

### Energy metabolism indicators

**Figure 2:**
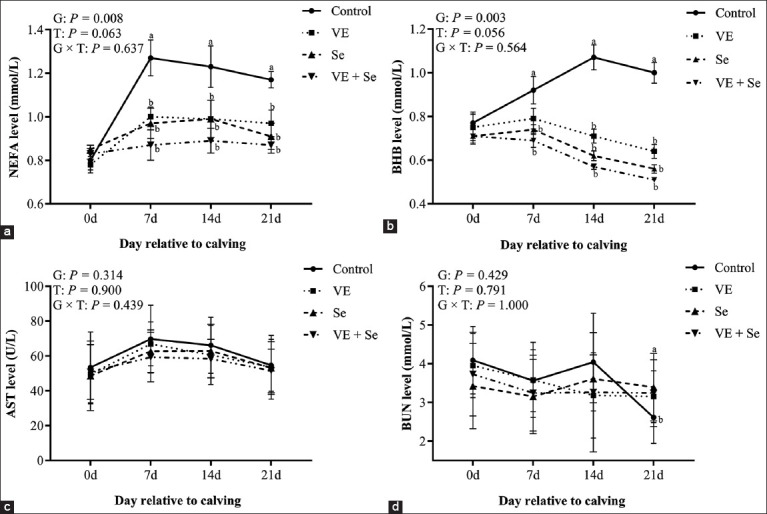
(a–d) Plasma energy metabolism index levels in the four experimental groups. Control (n = 10), VE (n = 10), Se (n = 10), and VE + Se (n = 10) groups. Different lowercase letters (a, b, and c) indicate statistically significant differences at p < 0.05, T=Time effect, G=Between-group effect, G × T=Inter-group interaction with time, respectively, VE=Vitamin E, Se=Selenium.

Figure 2 shows the effects of supplementation on NEFA, BHB, AST, and BUN levels. A significant intergroup effect was observed for NEFA and BHB (p < 0.05), but no significant differences were found for AST and BUN (p > 0.05). NEFA and BHB levels were significantly reduced at all time points in the VE, Se, and VE + Se groups compared to the control (p < 0.05), with no group × time interaction observed. AST levels remained unaffected by supplementation. BUN levels were significantly higher in the VE + Se group at 21 days post-partum compared to the control (p < 0.05), with no significant changes at other time points.

### Oxidative stress biomarkers

**Figure 3:**
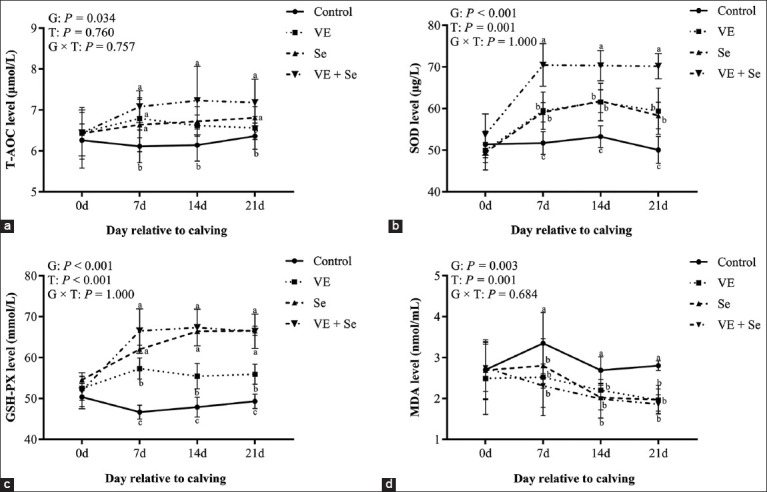
(a–d) Plasma oxidative stress index levels in the four experimental cow groups. Different lowercase letters (a, b, and c) indicate statistically significant differences at p < 0.05, T=Time effect, G=Between-group effect, G × T=Inter-group interaction with time, respectively.

Figure 3 illustrates the impact of VE and Se on oxidative stress markers. Supplementation significantly increased T-AOC, SOD, and GSH-Px levels and reduced MDA concentrations (p < 0.05). A significant time effect was also observed for SOD, GSH-Px, and MDA (p < 0.05), though no group × time interaction was detected. The VE + Se group exhibited the most substantial improvement in antioxidant status, with significantly higher SOD and GSH-Px levels than the individual VE or Se groups at all measured time points.

### Immune function indicators

**Figure 4:**
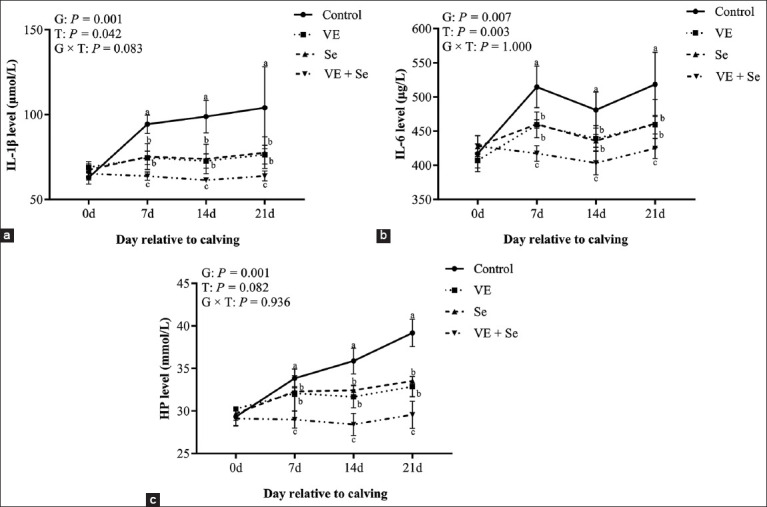
(a–c) Plasma immune function levels in the four experimental groups. Different lowercase letters (a, b, and c) indicate statistically significant differences at p < 0.05, T=Time effect, G=Between-group effect, G × T=Inter-group interaction with time, respectively.

Figure 4 shows changes in IL-1β, IL-6, and HP levels. Significant group and time effects were observed for IL-1β and IL-6 (p < 0.05), while HP was significantly affected by group only. All three biomarkers were significantly reduced in the supplemented groups compared to the control (p < 0.05), with the VE + Se group showing the lowest levels. No significant group × time interaction was found.

### Reproductive performance and disease incidence

Reproductive metrics and post-partum disease incidence are summarized in Tables 1 and 2. No significant differences were noted among groups for days to first estrus, first service, or number of artificial inseminations (p > 0.05). However, the VE + Se group had a significantly lower incidence of metritis compared to the control (p < 0.05), while mastitis incidence was significantly reduced in the Se and VE + Se groups. All three supplemented groups exhibited a significantly lower incidence of ketosis than the control (p < 0.05).

**Table 2:** Reproductive performance of the four experimental cow groups.

Item	Groups			

	Control (n = 10)	VE (n = 10)	Se (n = 10)	VE + Se (n = 10)
Age at first estrus (day)	65.75 ± 2.94	63.96 ± 2.58	64.07 ± 2.13	63.59 ± 2.00
First service day (day)	67.85 ± 3.32	65.02 ± 3.24	66.58 ± 3.08	65.00 ± 3.08
Insemination (times)	2.33 ± 0.59	1.76 ± 0.52	1.73 ± 0.59	1.69 ± 0.55
Estrous rate (%)	70	80	80	80
Pregnancy rate (%)	60	70	70	80

VE=Vitamin E, Se=Selenium

### Correlation between plasma VE/Se levels and disease incidence

Tables 3–5 present Spearman correlation analyses showing that higher plasma VE and Se levels were associated with lower disease incidence. Mastitis incidence was significantly and negatively correlated with both VE (R = −0.677, p = 0.031) and Se (R = −0.798, p = 0.006) levels. Metritis was significantly correlated with Se (R = −0.696, p = 0.025) but not VE (p = 0.071). Although the correlation between VE levels and ketosis was not statistically significant, the trend remained negative.

**Table 3:** Disease prevalence in four groups of dairy cows.

Item (%)	Groups			

	Control (n = 10)	VE (n = 10)	Se (n = 10)	VE + Se (n = 10)
Metritis	30 (3/10)^a^	20 (2/10)^a^	20 (2/10)^a^	10 (1/10)^b^
Mastitis	40 (4/10)^a^	30 (3/10)^a^	20 (2/10)^b^	0^b^
Ketosis	10 (1/10)^a^	0	0	0

^a and b^indicate significant differences (p *<* 0.05). VE=Vitamin E, Se=Selenium

**Table 4:** Correlation between plasma vitamin E levels and disease incidence in experimental cows.

Item	R	p-value
Mastitis	− 0.677*	0.031
Metritis	− 0.593	0.071
Ketosis	− 0.548	0.101

R=Spearman’s rank correlation coefficient;

* p < 0.05;

**p < 0.01

**Table 5:** Correlation between plasma vitamin selenium level and disease incidence in experimental cows.

Item	R	p-value
Mastitis	− 0.798**	0.006
Metritis	− 0.696*	0.025
Ketosis	− 0.406	0.244

R=Spearman’s rank correlation coefficient;

* p < 0.05;

** p < 0.01

### Predictive value of VE and Se for disease risk

**Figure 5:**
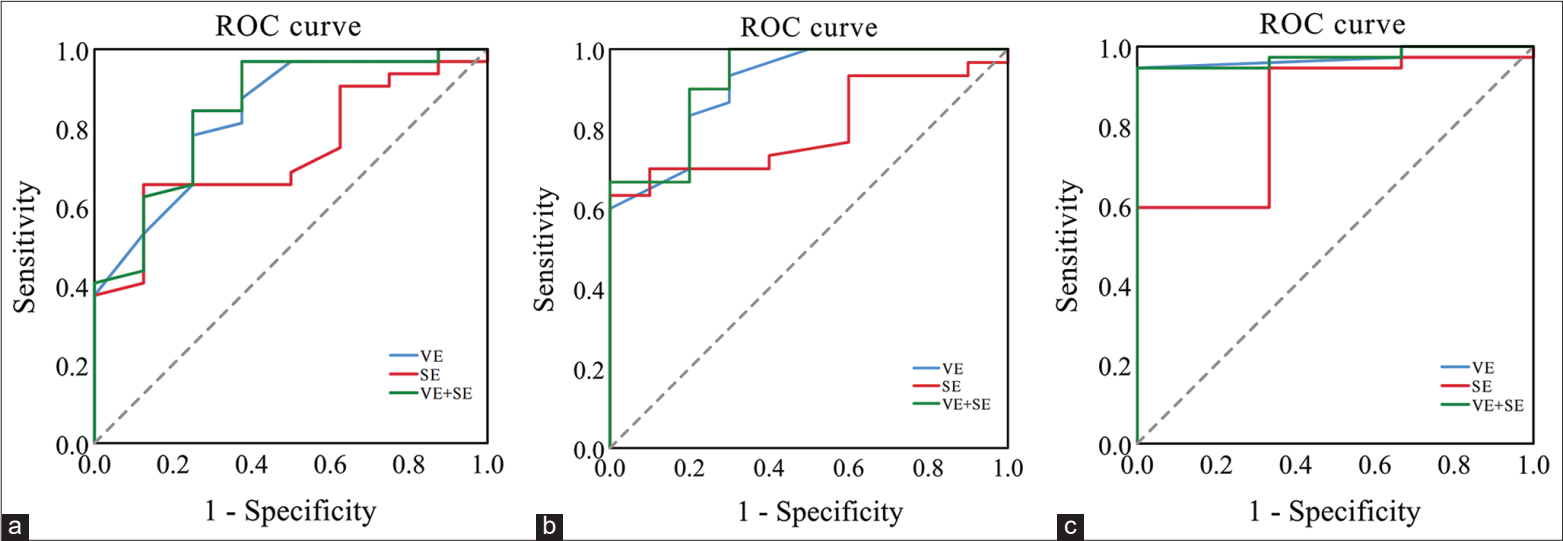
(a) ROC area under the curve of VE and Se in dairy cows with metritis. (b) ROC area under the curve of VE and Se in dairy cows with mastitis. (c) ROC area under the curve of VE and Se in dairy cows with ketosis. The AUC, sensitivity, and specificity values were calculated to evaluate diagnostic performance. Plasma VE and Se concentrations were measured in mg/L and µg/L, respectively. VE=Vitamin E, Se=Selenium, ROC=Receiver operating characteristic, AUC=Area under the curve.

Figure 5 and Table 6 detail the ROC curve analysis for VE and Se as predictive markers of mastitis, metritis, and ketosis.

**Table 6:** Cutoff point, sensitivity, specificity, standard error, and area under the receiver operating characteristic curve of VE and Se for diagnosing mastitis, metritis, and ketosis in dairy cows.

Disease	Parameter	Cutoff point (mmol/L)	Sensitivity (%)	Specificity (%)	AUC	SEM	p-value
Metritis	VE	0.45	78.1	75.0	0.834	0.078	0.004
	Se	4.21	65.6	87.5	0.736	0.086	0.041
	VE + Se	-	96.9	65.5	0.848	0.078	0.003
Mastitis	VE	0.45	83.3	80.0	0.908	0.051	<0.001
	Se	2.615	96.8	40.0	0.800	0.069	0.005
	VE + Se	-	90.0	80.0	0.923	0.048	<0.001
Ketosis	VE	0.05	94.6	33.3	0.973	0.025	0.007
	Se	2.21	91.9	66.7	0.838	0.109	0.054
	VE + Se	-	97.3	66.7	0.973	0.026	0.007

VE=Vitamin E, Se=Selenium, AUC=Area under the curve, SEM=Standard error of the mean

- For metritis, VE had an area under the curve (AUC) of 0.834, Se had an AUC of 0.736, and combined VE + Se had an improved AUC of 0.848
- For mastitis, VE yielded an AUC of 0.908, Se an AUC of 0.800, and their combination increased the AUC to 0.923
- For ketosis, VE alone had an AUC of 0.973, with a sensitivity of 94.6% and specificity of 33.3%. Se alone had an AUC of 0.838. The combined VE + Se model retained an AUC of 0.973 but improved specificity (66.7%) and sensitivity (97.3%).

These findings demonstrate that plasma VE and Se concentrations are valuable for early disease risk prediction, particularly when used together.

### Economic benefit analysis

Table 7 summarizes the economic returns for each treatment group. All three supplementation groups showed increased milk yield compared to the control, resulting in higher daily net profits. The VE + Se group demonstrated the greatest economic gain, followed by the VE group and then the Se group. These results indicate that combined supplementation not only improves health and performance outcomes but also provides the greatest return on investment under field conditions.

**Table 7:** Economic benefits of four dairy herd trials.

Item	Groups			

	Control (n = 10)	VE (n = 10)	Se (n = 10)	VE + Se (n = 10)
Milk income (¥/day)	118.93	132.41	127.51	137.52
Milk price (¥/kg)	3.50	3.50	3.50	3.50
Milk yield (kg/day)	33.98	37.83	36.43	39.29
Increased economic benefits (¥/day)	0	13.48	8.58	18.59
Increased net profit (¥/day)	0	13.31	8.54	18.38
Feed cost (¥)	25.00	25.00	25.00	25.90
Labor cost (¥)	7.50	7.50	7.50	7.50
Vet cost saving (¥)	0	+5.00	+6.00	+8.00
Supplement cost (¥)	0	0.17	0.04	0.21
Net profit/day (¥)	86.43	104.74	101.97	111.91
Net profit/305 days of lactation (¥)	26161.50	31946.70	31101.15	34130.55

VE=Vitamin E, Se=Selenium

## DISCUSSION

### Importance of VE and Se in transition cows

VE and Se are essential micronutrients in dairy cows, particularly during the transition period, where their roles in supporting physiological function, antioxidant defense, and immune modulation become critical. Deficiencies in either VE or Se are common in transition cows and are associated with increased disease susceptibility and reduced productivity [28, 29]. In the current study, supplementation with VE and Se effectively elevated their plasma concentrations in the treated groups, with the VE + Se combination achieving the most pronounced increases. These outcomes align with previous reports showing that co-supplementation of Se with iodine and cobalt [30] or VE [31] can improve milk yield. In our study, milk yield was significantly higher in the VE + Se group than in the control, indicating enhanced production performance due to improved nutritional support.

### Effects on energy metabolism and organ function

During the transition period, dairy cows frequently experience negative energy balance due to high milk production demands and insufficient DM intake [32]. This imbalance promotes the mobilization of NEFA, which are oxidized to ketone bodies such as BHB [33]. Our study demonstrated that VE and Se supplementation significantly reduced NEFA and BHB levels in all treated groups without affecting liver (AST) or kidney (BUN) function. The VE + Se group showed a slightly elevated BUN at 21 days post-partum, potentially reflecting enhanced protein turnover rather than renal dysfunction. These findings confirm the role of antioxidant supplementation in alleviating metabolic stress, consistent with a previous study by Chalmeh *et al*. [34].

### Antioxidant enhancement through VE and Se supplementation

VE acts as a chain-breaking antioxidant that neutralizes lipid peroxides, while Se functions as a cofactor for GSH-Px [35]. Supplementation with these antioxidants increased T-AOC, SOD, and GSH-Px levels, while significantly reducing MDA, a marker of lipid peroxidation. These trends were most pronounced in the VE + Se group. Similar results were reported by Somagond *et al*. [22] and Zhang *et al*. [36], who observed improved antioxidant responses following injectable trace minerals. The positive correlation between serum Se and GSH-Px observed by Wang *et al*. [37] also aligns with our findings, further supporting the efficacy of combined antioxidant strategies in periparturient cows.

### Immunomodulatory and anti-inflammatory effects

In addition to antioxidant defense, VE and Se modulate immune responses. Our study showed that plasma levels of pro-inflammatory cytokines IL-1β and IL-6, and the acute phase protein HP, were significantly reduced following supplementation, particularly in the VE + Se group. Previous studies by Somagond *et al*. [22] have shown that Se-containing micronutrients lower cytokine expression in cows, and deficiencies in Se are linked to elevated inflammatory responses in multiple tissues [38]. Furthermore, VE and Se have been shown to mitigate inflammation induced by *Staphylococcus aureus* through downregulation of IL-1β, IL-6, and tumor necrosis factor alpha [39]. These observations suggest that VE and Se exert synergistic effects in modulating inflammatory pathways and enhancing immune resilience.

### Reproductive health and performance

Reproductive inefficiency in dairy cows can stem from negative energy balance, uterine infections, and oxidative stress [40–42]. Although no statistically significant differences in reproductive parameters were observed in this study, cows receiving VE + Se exhibited numerically improved performance in estrus onset and insemination efficiency. Past research demonstrated improved pregnancy and lambing rates with VE/Se injection in ewes [43], and enhanced estrus expression in dairy cows [10]. Thus, even modest improvements in reproductive indices, when combined with better metabolic and immune status, support the use of antioxidant supplementation as part of transition cow management.

### Reduction in disease incidence

VE and Se supplementation significantly reduced the incidence of ketosis, mastitis, and metritis in treated groups. The lowest disease rates were observed in the VE + Se group. Spearman correlation analysis confirmed negative associations between plasma VE and Se levels and disease incidence, especially for mastitis and metritis. These findings are consistent with previous studies by Sun *et al*. [5], Pecoraro *et al*. [6], Sun *et al*. [19], Li *et al*. [20], Wang *et al*. [37], and Arshad *et al*. [44] that demonstrated the protective effects of VE and Se against metabolic and infectious disorders during the transition period. The observed improvements in immune function and antioxidant status likely contributed to enhanced disease resistance.

### Diagnostic value of VE and Se as predictive markers

ROC analysis revealed that plasma VE and Se concentrations had significant predictive value for common transition-period diseases. For metritis, VE alone yielded an AUC of 0.834, Se alone 0.736, and their combination 0.848. Mastitis prediction was even stronger (AUC of 0.923 with combined supplementation), and ketosis prediction reached an AUC of 0.973. Combined supplementation not only improved sensitivity and specificity but also extended the diagnostic utility of these biomarkers. These results highlight the potential of plasma VE and Se monitoring as an early warning system for transition cow health management.

### Economic outcomes and profitability

Economic analysis indicated that all supplementation groups generated higher net returns compared to the control group, with the VE + Se group achieving the greatest profitability. The cost of supplementation was modest (¥ 0.17/day for VE and ¥ 0.04/day for Se), and the VE + Se group achieved a net profit increase of ¥18.38/day over the control. These results underscore the economic justification for incorporating targeted micronutrient supplementation during the transition period to enhance milk production and reduce disease-associated losses.

### Study limitations and future directions

This study has several limitations. The relatively small sample size limits generalizability, and the short post-partum follow-up period precluded evaluation of long-term health or fertility outcomes. In addition, the unblinded study design introduces potential observer bias. Finally, no molecular analyses were performed to elucidate the underlying regulatory mechanisms of observed effects. Future research should include larger sample sizes, extended monitoring periods, and molecular assays (e.g., gene expression, cytokine signaling pathways) to better characterize the mechanistic interactions of VE and Se in dairy cows.

## CONCLUSION

This study demonstrated that VE and Se supplementation, particularly when combined, offers substantial benefits to transition dairy cows. The VE + Se group exhibited significantly elevated plasma levels of both nutrients, improved antioxidant enzyme activity (↑T-AOC, SOD, GSH-Px; ↓MDA), and reduced concentrations of pro-inflammatory cytokines (↓IL-1β, IL-6, HP), indicating enhanced oxidative balance and immune resilience. Supplementation also led to a marked reduction in the incidence of transition-period diseases–ketosis, mastitis, and metritis–while improving energy metabolism (↓NEFA, BHB) without negatively impacting liver or kidney function.

A novel finding of this study is the predictive diagnostic value of plasma VE and Se levels. ROC analysis showed that combined VE and Se concentrations could effectively identify cows at risk for mastitis (AUC = 0.923), metritis (AUC = 0.848), and ketosis (AUC = 0.973), offering a feasible early warning tool for herd health monitoring. From an economic perspective, VE + Se supplementation yielded the greatest net profit, with a daily return of ¥ 18.38 per cow over the control group, confirming its practical value in commercial dairy operations.

The strength of this study lies in its comprehensive approach–simultaneously assessing antioxidant function, immune response, disease prevalence, production performance, diagnostic potential, and economic return. These integrated insights provide a robust scientific foundation for including VE and Se in transition cow nutrition strategies.

In conclusion, strategic supplementation with VE and Se during the transition period enhances cow health, improves productivity, and reduces disease-associated economic losses. The combined use of VE and Se proves to be more effective than individual supplementation, offering a practical, evidence-based solution for optimizing transition cow management. Future studies should focus on long-term outcomes, reproductive efficiency, and mechanistic pathways to further validate and expand these findings.

## DATA AVAILABILITY

All the generated data are included in the manuscript.

## AUTHORS’ CONTRIBUTIONS

YD, RS and CX: Study conception and design. RS, XJ, YH, YB, CX, YS, XJ and YD: Performed material preparation, data collection and analysis, and revised the manuscript. YD: Drafted the manuscript. All authors have read and approved the final version of the manuscript.

## References

[1] Van Q.C.D, Knapp E, Hornick J.L, Dufrasne I (2020). Influence of days in milk and parity on milk and blood fatty acid concentrations, blood metabolites and hormones in early lactation Holstein cows. Animals (Basel).

[2] Bronzo V, Lopreiato V, Riva F, Amadori M, Curone G, Addis M.F, Cremonesi P, Moroni P, Trevisi E, Castiglioni B (2020). The role of innate immune response and microbiome in resilience of dairy cattle to disease:The mastitis model. Animals (Basel).

[3] Yang S, Zhang H, Li F, Guo L (2025). Immunosuppression and nutritional regulation in periparturient dairy cows. Chin. J. Anim. Nutr.

[4] Olthof L.A, Domecq J.J, Bradford B.J (2023). Analysis of Jersey versus Holstein breed profitability on North Central US dairies. JDS Commun.

[5] Sun C, Shan F, Liu M, Liu B, Zhou Q, Zheng X, Xu X (2022). High-fat-diet-induced oxidative stress in giant freshwater prawn (*Macrobrachium rosenbergii*) via NF- B/NO signal pathway and the amelioration of vitamin E. Antioxidants (Basel).

[6] Pecoraro B.M, Leal D.F, Frias-De-Diego A, Browning M, Odle J, Crisci E (2022). The health benefits of selenium in food animals:A review. J. Anim. Sci. Biotechnol.

[7] Spears J.W, Weiss W.P (2008). Role of antioxidants and trace elements in health and immunity of transition dairy cows. Vet. J.

[8] Politis I (2012). Reevaluation of vitamin E supplementation of dairy cows:Bioavailability, animal health and milk quality. Animal.

[9] Harrison J.H, Hancock D.D, Conrad H.R (1984). Vitamin E and selenium for reproduction of the dairy cow. J. Dairy Sci.

[10] Khatti A, Mehrotra S, Patel P.K, Singh G, Maurya V.P, Mahla A.S, Chaudhari R.K, Narayanan K, Das G.K, Singh M, Sarkar M, Kumar H, Krishnaswamy N (2017). Supplementation of vitamin E, selenium and increased energy allowance mitigates the transition stress and improves postpartum reproductive performance in the crossbred cows. Theriogenology.

[11] Miyazawa T, Burdeos G.C, Itaya M, Nakagawa K, Miyazawa T (2019). Vitamin E:Regulatory redox interactions. IUBMB Life.

[12] Politis I, Hidiroglou M, Batra T.R, Gilmore J.A, Gorewit R.C, Scherf H (1995). Effects of vitamin E on immune function of. dairy cows. Am. J. Vet. Res.

[13] Keshri A, Bashir Z, Kumari V, Prasad K, Joysowal M, Singh M, Singh D, Tarun A, Shukla S (2019). Role of micronutrients during peri-parturient period of dairy animals:A review. Biol. Rhythm Res.

[14] Zou Y, Shao J, Li Y, Zhao F.Q, Liu J.X, Liu H (2019). Protective effects of inorganic and organic selenium on heat stress in bovine mammary epithelial cells. Oxid. Med. Cell. Longev.

[15] Chan J.M, Darke A.K, Penney K.L, Tangen C.M, Goodman P.J, Lee G.M, Sun T, Peisch S, Tinianow A.M, Rae J.M, Klein E.A, Thompson I.M, Kantoff P.W, Mucci L.A (2016). Selenium- or vitamin E-related gene variants, interaction with supplementation, and risk of high-grade prostate cancer in SELECT. Cancer Epidemiol. Biomarkers Prev.

[16] Sordillo L.M (2013). Selenium-dependent regulation of oxidative stress and immunity in periparturient dairy cattle. Vet. Med. Int.

[17] Youcef M, Djoher I (2016). Selenium in cattle:A review. Molecules.

[18] Hefnawy A.E.G, Tórtora-Pérez J.L (2010). The importance of selenium and the effects of its deficiency in animal health. Small Rumin. Res.

[19] Sun L, Liu G, Xu D, Wu Z, Ma L, Victoria S.M, Baumgard L.H, Bu D (2021). Milk selenium content and speciation in response to supranutritional selenium yeast supplementation in cows. Anim. Nutr.

[20] Li Y, Liu J.X, Xiong J.L, Wang Y.M, Zhang W.X, Wang D.M (2019). Effect of hydroxyselenomethionine on lactation performance, blood profiles, and transfer efficiency in early-lactating dairy cows. J. Dairy Sci.

[21] Xiao J, Khan M.Z, Ma Y, Alugongo G.M, Ma J, Chen T, Khan A, Cao Z (2021). The antioxidant properties of selenium and vitamin E;their role in periparturient dairy cattle health regulation. Antioxidants (Basel).

[22] Somagond Y.M, Alhussien M.N, Dang A.K (2023). Repeated injection of multivitamins and multiminerals during the transition period enhances immune response by suppressing inflammation and oxidative stress in cows and their calves. Front. Immunol.

[23] Zhang S, Li J, Li C, Wang J, Wang Z, Ding X, Bai S (2021). Effects of vitamin E supplementation on the health and productivity of dairy cows:A meta-analysis. Trop. Anim. Health Prod.

[24] NRC (2001). Nutrient Requirements of Dairy Cattle.

[25] Serrenho R.C, Williamson M, Berke O, LeBlanc S.J, DeVries T.J, McBride B.W, Duffield T.F (2022). An investigation of blood, milk, and urine test patterns for the diagnosis of ketosis in dairy cows in early lactation. J. Dairy Sci.

[26] Bleul U, Sacher K, Corti S, Braun U (2006). Clinical findings in 56 cows with toxic mastitis. Vet. Rec.

[27] Bogado Pascottini O, LeBlanc S.J, Gnemi G, Leroy J.L.M.R, Opsomer G (2023). Genesis of clinical and subclinical endometritis in dairy cows. Reproduction.

[28] Meglia G.E, Johannisson A, Petersson L, Waller K.P (2001). Changes in some blood micronutrients, leukocytes and neutrophil expression of adhesion molecules in periparturient dairy cows. Acta Vet. Scand.

[29] Weiss W.P, Todhunter D.A, Hogan J.S, Smith K.L (1990). Effect of duration of supplementation of selenium and vitamin E on periparturient dairy cows. J. Dairy Sci.

[30] Cook J.G, Green M.J (2010). Milk production in early lactation in a dairy herd following supplementation with iodine, selenium and cobalt. Vet. Rec.

[31] Eulogio G.L.J, Alberto S.O.J, Hugo V.C, Antonio C.N, Alejandro C.I, Juan M.Q (2012). Effects of selenium and vitamin E in the production, physicochemical composition and somatic cell count in milk of Ayrshire cows. J. Anim. Vet. Adv.

[32] Drackley J.K (1999). ADSA Foundation Scholar Award. Biology of dairy cows during the transition period:The final frontier?. J. Dairy Sci.

[33] Dong J, Loor J.J, Zuo R, Chen X, Liang Y, Wang Y, Shu X, Sun X, Jia H, Liu G, Wang Z, Li X, Li X (2019). Low abundance of mitofusin 2 in dairy cows with moderate fatty liver is associated with alterations in hepatic lipid metabolism. J. Dairy Sci.

[34] Chalmeh A, Pourjafar M, Badiei K, Mirzaei A, Jalali M, Mazrouei Sebdani M (2021). Effects of dietary antioxidants on glucose and insulin responses to glucose tolerance test in transition dairy cows. Domest. Anim. Endocrinol.

[35] Smith K.L, Hogan J.S, Weiss W.P (1997). Dietary vitamin E and selenium affect mastitis and milk quality. J. Anim. Sci.

[36] Zhang L, Wang Z, Zhou P, Fu L, Zhang L, Xu C, Loor J.J, Zhang T, Chen Y, Zhou Z, Dong X (2022). Vitamin E supplementation improves post-transportation systemic antioxidant capacity in yak. PLoS One.

[37] Wang D, Jia D, He R, Lian S, Wang J, Wu R (2020). Association between serum selenium level and subclinical mastitis in dairy cattle. Biol. Trace Elem. Res.

[38] Inal M.E, Kanbak G, Sunal E (2001). Antioxidant enzyme activities and malondialdehyde levels related to aging. Clin. Chem. Acta.

[39] Khan M.Z, Ma Y, Xiao J, Chen T, Ma J, Liu S, Wang Y, Khan A, Alugongo G.M, Cao Z (2022). Role of selenium and vitamins E and B9 in the alleviation of bovine mastitis during the periparturient period. Antioxidants (Basel).

[40] Serbetci I, González-Grajales L.A, Herrera C, Ibanescu I, Tekin M, Melean M, Magata F, Malama E, Bollwein H, Scarlet D (2024). Impact of negative energy balance and postpartum diseases during the transition period on oocyte quality and embryonic development in dairy cows. Front. Vet. Sci.

[41] Barker A.R, Schrick F.N, Lewis M.J, Dowlen H.H, Oliver S.P (1998). Influence of clinical mastitis during early lactation on reproductive performance of Jersey cows. J. Dairy Sci.

[42] Paiano R.B, Bonilla J, Pugliesi G, Moreno A.M, Baruselli P.S (2023). Evaluation of clinical and subclinical endometritis impacts on the reproductive performance and milk production of dairy cows in Brazilian herds. Reprod. Domest. Anim.

[43] Awawdeh M.S, Eljarah A.H, Ababneh M.M (2019). Multiple injections of vitamin E and selenium improved the reproductive performance of estrus-synchronized Awassi ewes. Trop. Anim. Health Prod.

[44] Arshad M.A, Ebeid H.M, Hassan F.U (2020). Revisiting the effects of different dietary sources of selenium on the health and performance of dairy animals:A review. Biol. Trace Elem. Res.,.

